# Isolation and Antibiotic Resistant Research of *Tetragenococcus halophilus* from Xuanwei Ham, A China High-Salt-Fermented Meat Products

**DOI:** 10.3390/antibiotics8030151

**Published:** 2019-09-16

**Authors:** Yinjiao Li, Luying Shan, Chen Zhang, Zhan Lei, Ying Shang

**Affiliations:** 1 Yunnan Institute of Food Safety, Kunming University of Science and Technology, Yunnan 650500, China; 18487156903@163.com (Y.L.); shanluying1994@163.com (L.S.); chaserzc09@163.com (C.Z.); lz1135300654@163.com (Z.L.); 2 Faculty of agriculture and food, Kunming University of Science and Technology, Yunnan 650500, China

**Keywords:** Xuanwei ham, *Tetragenococcus halophilus*, antibiotic resistant, antibiotic resistance genes

## Abstract

We assessed the prevalence of antibiotic resistant and antibiotic resistance genes for 49 *Tetragenococcus halophilus* (*T. halophilus*) strains isolated from Xuawei ham in China. The antibiotic resistance phenotype was detected by the Bauer–Kirby (K–B) method and the results showed that 49 isolates can be considered completely susceptible to penicillin, ampicillin, amoxicillin, cefradine, cefotaxime, tetracyclines, minocycline, doxycycline, and vancomycin, but resistant to gentamicin, streptomycin, neomycin, polymyxinB, cotrimoxazole. This resistance was sufficiently high to consider the potential for acquisition of transmissible determinants. A total of 32 isolates were resistant to ofloxacin, 4 isolates were resistant to ciprofloxacin and chloramphenicol, and 2 isolates were resistant to ceftazidime and ticarcillin. The antibiotic resistance genes were detected by routine polymerase chain reaction (PCR). Among the 26 antibiotic resistance genes, 5 varieties of antibiotic resistance genes, including *acrB*, *blaTEM*, *AAda1*, *SulII*, and *GyrB* were detected and the detection rates were 89.79%, 47.7%, 16.33%, 77.55%, and 75.51%, respectively. The potential acquisition of transmissible determinants for antibiotic resistance and antibiotic resistance genes identified in this study necessitate the need for a thorough antibiotic resistance safety assessment of *T. halophilus* before it can be considered for use in food fermentation processes.

## 1. Introduction

*Tetragenococcus halophilus*, formerly classified as *Pediococcus halophilus* and subsequently reclassified as *Tetragenococcus* by Collins et al. [1], is a gram-positive, non-spore quadruple sphere, exopolysaccharide, nonmotile, and facultative anaerobic, and the final product of glucose metabolism is lactic acid without gas formation [2,3]. *T. halophilus* has been widely detected in various salted and fermented food such as fermented fish products, soy pastes, soy sauce, and is considered a potential starter for their production to improve the quality or to shorten the fermentation cycle and so on [4,5,6,7,8,9,10]. However, there is hardly any literature report on the isolation and identification of *T. halophilus* from salt-cured and fermented Xuanwei ham.

*T. halophilus* as a potential starter of this species, which not only improves the sensory properties and the flavor, as well as effectively reducing harmful substances, but also possess health functionality in fermented foods. Adding *T. halophilus* in the fermentation process of fish sauce can significantly increase the content of total amino acids and free amino acids and increase the content of umami amino acids, such as glutamic acid [10]. The content of volatile substances, such as 2-methylpropanal, 2-methylbutanal, 3-methylbutanal, and benzaldehyde, which have a positive influence on the flavor, is also significantly increased in the fish sauce to which the *T. halophilus* is added [11]. The yeast-derived fermentation metabolites combined with the metabolites of the *T. halophilus* can significantly increase the content of esters in the product and improve the flavor of the product [12]. Combining of *T. halophilus* and yeast as a starter fermented soybean meal can reduce the fermentation cycle in addition to the volatile flavor component [13]. Similarly, studies by Satomi, Jeong, Kuda, and others have shown that suitable strains of *T. halophilus* can reduce the production of certain biogenic amines in fermented foods [10,11,14]. In addition, it has been reported that *T. halophilus* can play a probiotic role in promoting health. Masuda and other studies have shown that the *T. halophilus* Th221 strains isolated from soy sauce helped to increase the Th1-type immune response, improved perennial allergic rhinitis (PAR) symptoms, and had the potential for improving allergy symptoms [15].

Fermented foods have a long history of human consumption and lactic acid bacteria (LAB) are ubiquitous in fermented foods. This long history of human exposure and consumption has led to the reasonable conclusion that they are generally safe. However, increasing issues of human infections are reportedly caused by LAB [16]. Additionally, it has been reported that probiotics produce harmful metabolic activities and drug resistance shifts [17,18]. These results meant that the organisms are no longer automatically considered safe. Antibiotic resistance has become a main burden of public health worldwide and food is an important carrier for effectively transferring antibiotic resistant factors into the intestinal tract of consumers [19,20]. At present, there are many documents documenting that antibiotic resistant factors can be further transferred to humans through the food chain and livestock [21]. A number of studies have been performed to assess the antibiotic resistance of species of the genera *Enterococcus*, *Lactobacillus*, and *Lactococcus* from fermented foods, but there is little literature that studies the drug resistance of *T. halophilus*. At present, whether they carry antibiotic resistance genes has become an important indicator for evaluating the safety of a strain and for determining whether it can be used as a safe strain for food.

Therefore, based on the discovery of this vacancy, this study screened and identified the *T. halophilus* from Xuanwei ham, providing a basis for the flavor research, the safety, and the innovation of the fermented food starter of Xuanwei ham. Based on our previous study, the dominant bacterial community in Xuanwei ham is *Tetragenococcus*. The dominance in Xuanwei ham suggests its potential as a starter culture for the mass production of this product. Therefore, in this study, we used selective medium for separation and enrichment culture to screen for *T. halophilus* and identified the isolates by combining traditional and modern molecular biology methods to evaluate the antibiotic resistance of *T. halophilus*, including its resistant phenotype and resistance genes.

## 2. Results

### 2.1. Screening T. Halophilus Isolates by GroEL Gene and 16S rRNA Gene

**Figure 1:**
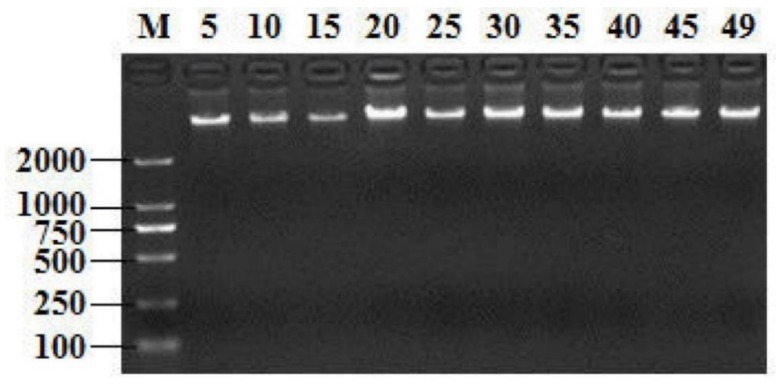
Partial electropherogram result of genomic DNA of isolated strains. M: DNA Maker DL2000; Lanes 5–49: DNA bands of 10 isolated strains.

**Figure 2:**
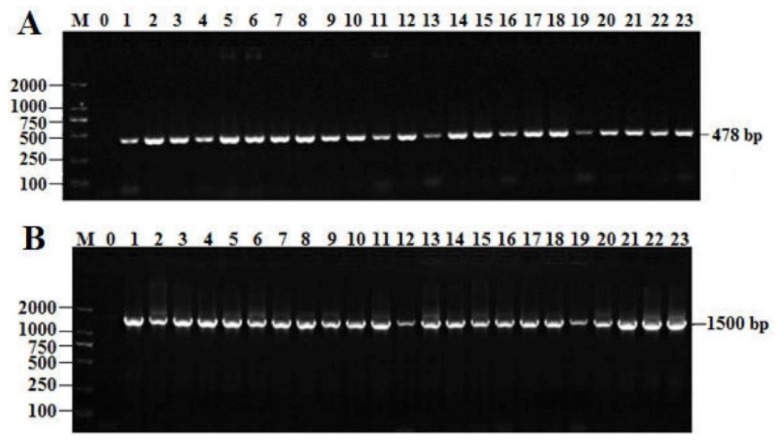
The PCR (polymerase chain reaction) results of the screened *T. halophilus* strains. (**A**) The screened *T. halophilus* strains by amplification of *groEL* gene; Lane M: DNA Marker DL2000; Lane 0: negative; Lane 1–23: Candidate strain of T. halophilus; (**B**) Electrophoresis of amplification product; Lane M: DNA Maker; Lane 0: Negative control; Lanes 1–23: The band of PCR amplification products of DNA primer PCR.

Extracting high-quality DNA from isolates is crucial for downstream molecular experiments. All the electropherograms showed the extracted DNAs with good quality; the partial results are shown in Figure 1. The PCR products of the *groEL* gene and the 16S rRNA gene amplified with primers were analyzed by 2% agarose gel, as shown in Figure 2. The *groEL* gene and16S rRNA gene amplification primers specifically extended the single target band to the isolated strains, which had no non-specific products and the background was clean. Hence, a total of 49 candidate strains were screened.

### 2.2. Identification of the Selected Bacteria

The sequence complements generated in this study were based primarily on BLAST search alignments and recent related literature. Raw sequences were assembled with BioEdit v. 7.2.5. Multiple sequence alignments were made using BioEdit 7.2 and ClustalX v.1. The alignments were examined visually and improved where necessary. Phylogenetic analyses of the combined aligned consisted of maximum likelihood (ML), MrBayes, and maximum parsimony (MP) analyses. Ambiguously aligned regions were removed from all analyses and gaps were treated as ‘‘missing data’’ in the phylogenetic analyses. Related sequences were selected to reveal the closest matching sequence to *T. halophilus*. A similarity to the 16S rRNA gene sequence of the *T. halophilus* was greater than 99% and was identified as a *T. halophilus*.

**Figure 3:**
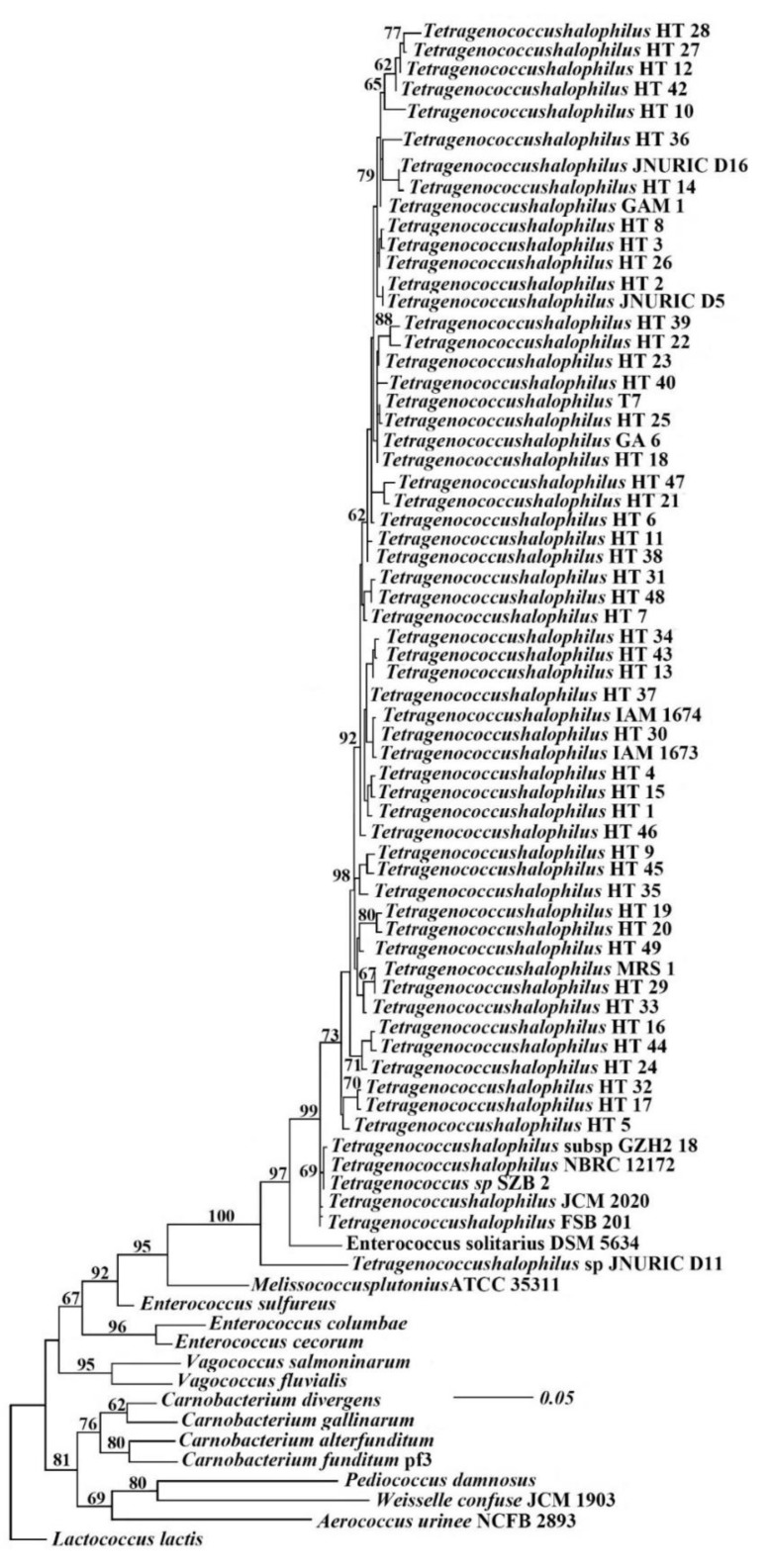
Phylogenetic analysis of 49 strains of *T halophilus* based on 16S rRNA sequence.

It can be seen from Figure 3 that the 49 isolates screened have 100% homology with *Tetragenococcus* sp. JNURIC D11 (GenBank: GQ150505.1) and they are clustered into the same branch with *T. halophilus* JCM2020 (GenBank: LC269262.1), *T. halophilus* NBRC12172 (GenBank: NR_076296.1), *T. halophilus* subsp. GZH2-18 (GenBank: MG654641.1), *Tetragenococcus* sp. JNURIC D16 (GenBank: GQ150510.1), *T. halophilus* MRS1 (GenBank: MK063722.1), and *T. halophilus* IAM1674 (GenBank: EU689055.1), and also have the closest relationship. From this, it can be seen that the 49 strains isolated from the Xuanwei ham are *T. halophilus* in the genus *Tetragenococcus*.

### 2.3. Experimental Results of Drug resistance Phenotype of 49 Isolates of T. Halophilus

A total of 19 antibiotic susceptibility tests were performed on 49 strains of *T. halophilus* using the K–B method. The size of the inhibition zone was explained by reference to the CLSI report method. It can be seen from Table 1 that the resistance rate of *T. halophilic* isolated from Xuanwei ham to gentamicin, streptomycin, neomycin, polymyxin B, and cotrimoxazole reached 100%, the resistance rate to ofloxacin reached 65.31%, the intermediate rate reached 30.61%, the resistance rate to ciprofloxacin and chloramphenicol was 8.16%, and the intermediate rate of ciprofloxacin was 24.49%. The intermediate rate of the chloramphenicol was 10.2%. The resistance rate to ticarcillin and ceftazidime was 4.08%, while the intermediate rate for ceftazidime was 10.2%, and there was susceptibility for ticarcillin, penicillin, ampicillin, amoxicillin, cefradine, cefotaxime, tetracyclines. The sensitivity rate of minocycline, doxycycline, and vancomycin reached 100% and the intermediate rate to amoxicillin was 4.08%.

**Figure 4:**
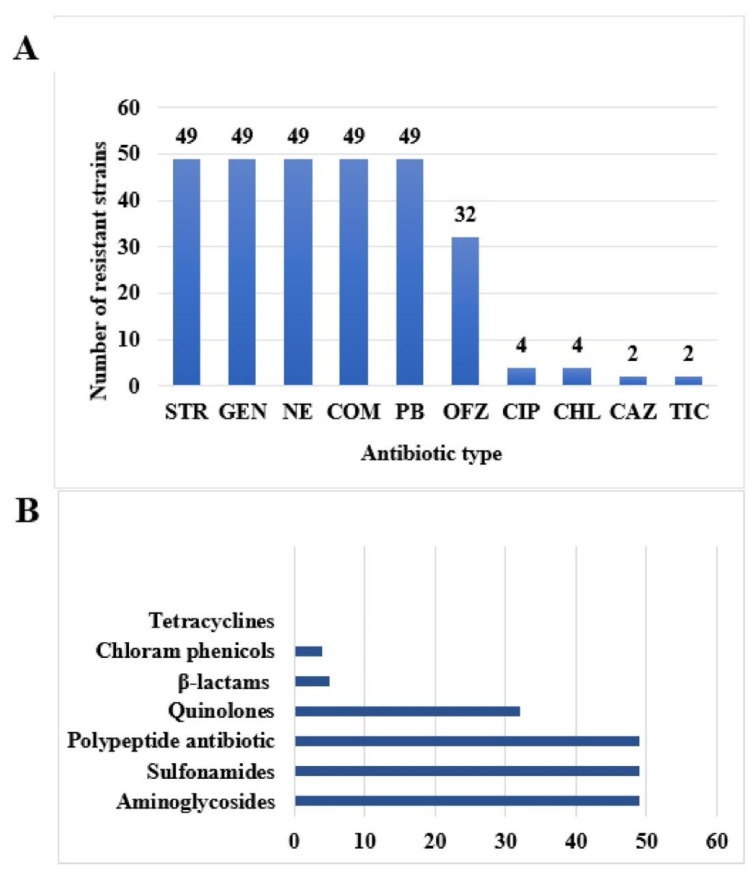
Distribution of drug resistance of 49 strains of *T. halophilus*. (**A**) Number of bacterial strains —by type of antibiotic; (**B**) Number of bacterial strains —by categories of antibiotic.

As can be seen from Figure 4A, 49 strains of *T. halophilus* are resistant to gentamicin, streptomycin, neomycin, polymyxin B, and sulfamethoxazole, 32 strains were resistant to ofloxacin, 4 strains are resistant to ciprofloxacin and chloramphenicol, and 2 strains are resistant to ticarcillin and ceftazidime. It can be seen from Figure 4B that 49 strains are mainly resistant to aminoglycosides, sulfonamides, polypeptide, and quinolone antibiotics, followed by β-lactams and chloramphenicol, and there was no resistance to tetracyclines, temporarily.

### 2.4. Drug Resistance Spectrum of 49 Strains of T. Halophilus

As shown in Table 2 and Table 3, the isolates of 49 strains of *T. halophilus* showed high concentration of drug resistance, with a total of 8 drug resistance spectra and no 0 or 9 resistant strains. The least number of resistant strains were 8 (only 6 strains), the most of which were 1, 2, 3, 4, and 5 (49 strains each, accounting for 100%), followed by 6 resistant strains (32 strains, accounting for 65.31%), most of which were concentrated in this range.

### 2.5. Prevalence of the AR Genes Among 49 Isolates of T. Halophilus

**Figure 5:**
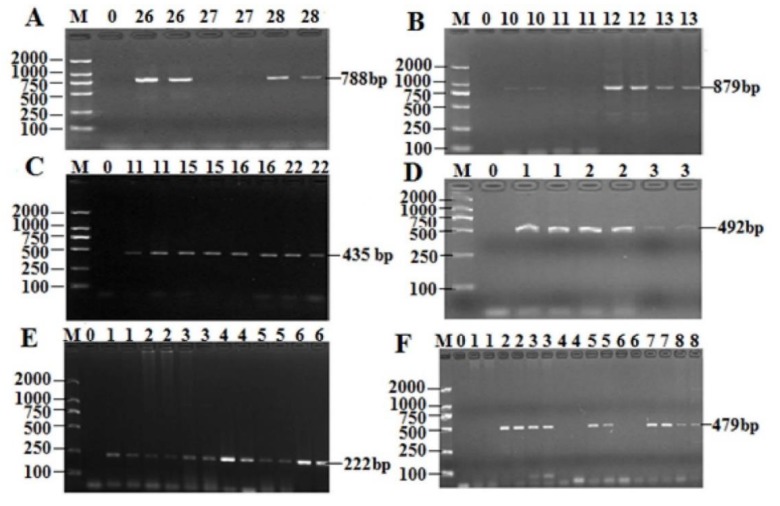
PCR detection results of some *T. halophilus* resistance genes. (**A**) *blaTEM* gene; (**B**) *GyrB* gene; (**C**) *SulII* gene; (**D**) *EmgrB* gene; (**E**) *acrB* gene; (**F**) *aadA1* gene; M: DL2000Marker, 0: Negative control.

All of the 49 strains of *T. halophilus* isolates isolated from Xuanwei ham were subjected to PCR detection of their AR gene patterns. The results are shown in Figure 5 and Table 4. Among the drug resistance genes, two drug resistance genes of aminoglycoside drugs were detected, the highest of which was *acrB*, with a positive rate of 89.79%. The positive detection rate of aadA1 was 28.57%. Only the TEM gene was detected in the *β*-lactams and the positive rate was 47.7%. As long as Sul2 was detected in the three genes of sulfa drugs, the positive rate was as high as 77.55%. Only the GyrB gene was detected in quinolones and the positive rate was 75.51%. The positive rate of EmgrB gene in polypeptide antibiotic was 61.22%. Among the 6 genes of tetracyclines, no drug resistance gene was detected. Chloramphenicol was also not detected in resistant genes.

### 2.6. Analysis of Drug Resistance Phenotype and Genotype Matching Rate of Isolates of T. Halophilus

**Figure 6:**
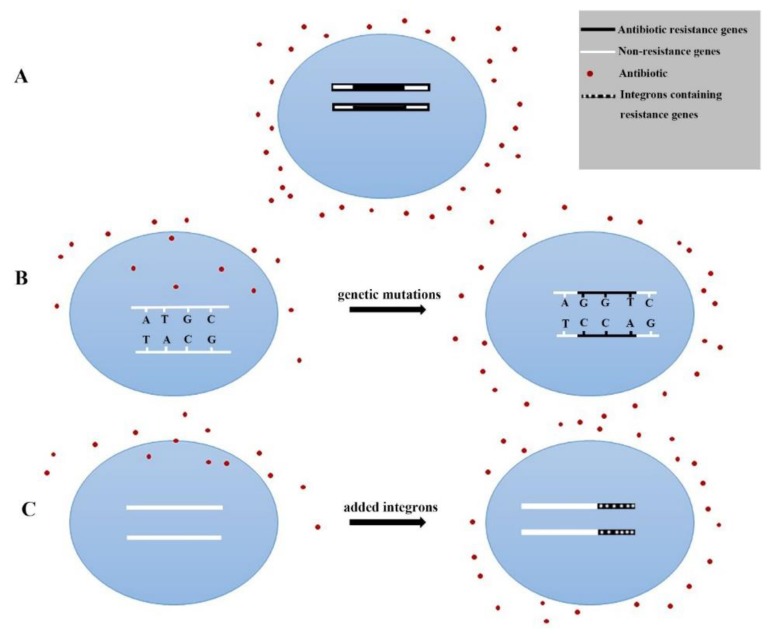
Gene mechanism. (**A**) Inherent drug resistance. Resistance genes exist in bacteria and are passed on from generation to generation. (**B**) Gene mutation or acquisition of new genes. Pressure to increase antibiotic production causes bacteria to mutate genes into drug resistance and, at the same time, bacteria can easily acquire resistance by ingesting resistance genes released after the death of another drug-resistant bacteria. (**C**) Integrons mediate drug resistance. Under the catalysis of integrase, integrons can capture and express exogenous genes, especially drug-resistant genes, so that drug-resistant genes can be transmitted between different species.

**Figure 7:**
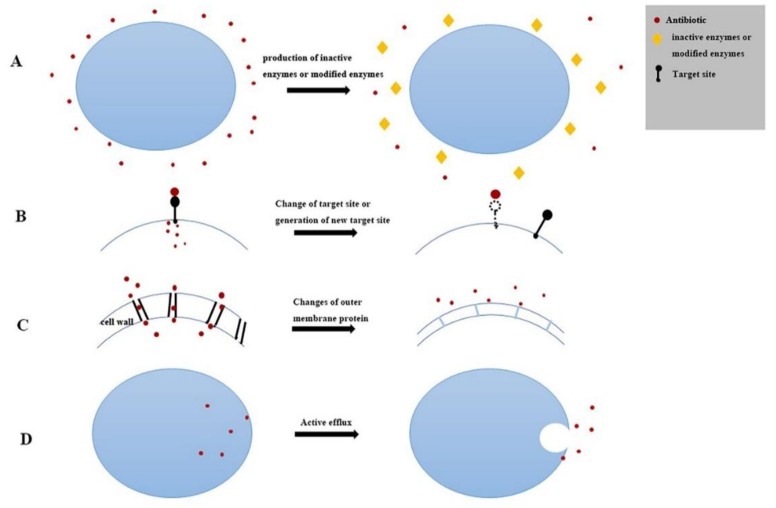
Biochemical (protein) mechanism. (**A**) Production of inactivated or modified enzymes. (**B**) Change of target site or generation of new target site. This makes is difficult for antibiotics to bind to cells, thus reducing the inhibitory effect of antibiotics. (**C**) Changes of outer membrane protein. Long-term drug effects can stimulate adventitia proteins to alter the cell wall structure and reduce permeability, thus hindering the penetration of antibiotics. (**D**) Active efflux system. When energy is available, membrane proteins selectively or non-selectively expel antibiotics from the cell, reducing drug concentration and leading to drug resistance.

Bacterial resistance to antibiotics is an antibiotic phenomenon in nature, but with the widespread use of antibiotics, bacterial resistance has gradually increased. The mechanism of bacterial drug resistance, which is commonly manifested in two aspects, genetic mechanism and biochemical (protein) mechanism, tends to be more complex. Figure 6 and Figure 7 show two resistance mechanisms.

The drug resistance of 49 isolates of *T. halophilus* was studied and the strains with the resistant phenotype and the resistant genes were detected for conformity analysis. As shown in Table 5, 49 isolates of *T. halophilus* were found to have resistance phenotypes and genotypes to tetracyclines, aminoglycosides, quinolones, sulfonamides, polypeptide antibiotic, and *β*-lactams, with the coincidence rates of 100%, 91.84%, 86.49%, 79.17%, 61.22%, and 62.5%, respectively. However, the resistant phenotype was detected for chloramphenicol and no drug resistance gene was detected.

Among the antimicrobial activities of bacteria, *β*-lactams is the most widely used drug in clinical applications. Its resistance mechanism is that bacteria produces *β*- lactamase or changes in PBPs, as shown in Figure 7A,B. Bacterial resistance to aminoglycosides and chloramphenics is mainly to produce inactivating enzymes, aminoglycoside inactivating enzymes including aminoglycoside acyltransferase, aminoglycoside adenosine transferase, and aminoglycoside phosphotransferase. A chloramphenicol inactivating enzyme is chloramphenicol acyltransferase (CAT), as shown in Figure 7A. For polypeptide vancomycin, tetracycline, quinolones, sulfonamides and aminoglycosides, drug resistance can also be induced by changing the target site, as shown in Figure 7B. On the other hand, the cell envelope and active efflux system are widespread in Gram-negative and Gram-positive bacteria (Figure 7C,D). In this study, for sulfonamides, aminoglycosides, chloramphenicol, and polypeptides, there were more resistant phenotype strains than resistant genotype strains. This may be influenced by resistance mechanisms at the biochemical (protein) level.

At the same time, genetic and biochemical mechanisms are mutually reinforcing. For example, most Gram-negative bacteria are resistant to polypeptide vancomycin. The reason for this is that it contains a resistance gene and an inactivating enzyme gene by itself or by gene mutation, shown in Figure 6A,B. As a consequence of the wide spread of resistance genes among different species of bacteria and the transformation of strains (inducing drug resistance) in the production of antibiotics, as shown in Figure 6B,C, a large number of resistant bacteria have increased.

After the sequenced results were compared with the reference sequences on GenBank, the homology of the above six antibiotic resistance gene sequencing results was more than 99%.

## 3. Discussion

Xuanwei ham is a high-salty and delicious dried ham cured by pickling, drying, and fermenting, while *T. halophilus* has been found in a variety of fermented foods and syrups. However, at present, there is almost no literature that reports on the screening of *T. halophilus* bacteria from Xuanwei ham. The *T. halophilus* has the potential effects of improving food flavor and increasing flavor substance content as a starter. So, in this study, 49 strains of *T. halophilus* was isolated from Xuanwei ham, which further enriched the microbial resources in the Xuanwei ham, and we assessed the antibiotic resistance safety of the 49 *T. halophilus* strains. The drug sensitivity results showed that the sensitivity rate of 49 isolates to penicillin, ampicillin, amoxicillin, cefradine, cefotaxime, tetracyclines, minocycline, doxycycline, and vancomycin reached 100%, which was roughly the same as the reported by Jeong et al. [22].

A total of 8 kinds of antibiotics were selected for drug sensitivity tests, which showed that almost all strains were sensitive to 5 kinds of antibiotics, including ampicillin, penicillin, tetracyclines, and vancomycin. The drug resistance rate of gentamicin and erythromycin were 11.54% and 3.85% respectively. All strains were resistant to ciprofloxacin and the drug resistance rate was up to 100%. All the strains in this study were resistant to gentamicin and the drug resistance rate was as high as 100%. The resistance rate to ciprofloxacin was 8.16%. In addition, the resistance rate of streptomycin, neomycin, polymyxin B, and compound xinomin reached 100%, ofloxacin reached 65.31%, chloramphenicol was 8.16%, and ticarcillin and ceftazidime was 4.08%.

The difference may be caused by the difference in the host of the *T. halophilus*. It has been reported that sulfonamides, aminoglycans, and quinolones have been widely used in pig and poultry production. In 2014, the World Health Organization (WHO) global surveillance report on antibiotic resistance showed evidence of a link between the use of antimicrobial agents in food animals and the emergence of resistance in common pathogens [23]. Teuber et al. stated, “The problem of drug resistance in human medicine will not be solved because resistant genes are constantly pouring into the human microflora through the food chain [24].” Berends et al. concluded that “most of the problems of drug-resistant bacteria in humans are related to the medical use of antimicrobials, particularly the limited impact of veterinary use [25].” However, these authors acknowledge that the impact of antibiotics as feed additives is extremely worrying. Therefore, it can be speculated that the antibiotic resistance of the *T. halophilic* isolate in Xuanwei ham may be mainly originated from the host. Pigs may only partially metabolize when they consume feed containing antibiotic additives or are injected with drugs containing antibiotics, and part of the antibiotics remain in the body.

On the other hand, the high concentration of the drug resistance spectrum indicates that there may be a problem of drug resistance gene transfer between these strains. At the same time, 26 common drug resistance genes of *T. halophilic* were detected. The drug-resistant phenotype and genotype of *T. halophilic* have a high coincidence rate, indicating that the drug-resistant phenotype of 49 isolates of *T. halophilus* isolates has a certain correlation with the drug resistance genes from themselves. Among them, some strains of aminoglycosides, sulfonamides, and chloramphenicol antibiotics detected the corresponding drug resistance phenotype, but no drug resistance genes related to the strain were detected. According to reports in the literature, when a bacterial strain has a drug-resistant phenotype to a certain antibiotic, but does not contain the corresponding drug-resistant gene, it can be inferred that the bacterial strain’s resistance to this antibiotic may be inherent resistance and the presence of a drug-resistant phenotype does not necessarily carry the corresponding drug-resistant gene [26]. Casado and Muñoz et al. tested the resistance of *Lactobacillus pentosus* and *Leuconostoc* to some antibiotics and found that no drug resistance genes were detected in strains with resistant phenotypes [27]. Zhang Hongmei et al. reported that 7 strains of LAB isolated from yoghurt were resistant to ampicillin and tetracyclines, but only 5 strains were detected that carried the ampicillin resistant gene Amp and no tetracyclines resistant gene was detected [28]. Hummel et al. found that some lactic acid bacteria strains showed low resistance to ampicillin, penicillin, chloramphenicol, and tetracyclines, but no known resistance genes were detected, although some strains have cat genes, none of these strains was phenotypically resistant to chloramphenicol, and these cat genes were silenced under both induction and non-induction conditions by reverse transcription PCR [29].

In this study, the resistance gene related to the isolated strain was detected in *β*-lactams and quinolone antibiotics, but the number of resistant phenotype strains was less than that of resistant genotype strains. It is possible that the strain carries drug resistance genes associated with it, but the drug resistance genes may exist but remain silent. Qin Yuxuan et al. tested the drug resistance of lactic acid bacteria isolated from commercially available yoghurt and found that all strains were sensitive to erythromycin and tetracyclines, but detected the corresponding drug resistance genes [30]. This once again proved that there is no drug resistance phenotype that may also carry a drug resistance gene, possibly because the drug resistance gene it carries is not expressed or expressed insufficiently.

At present, whether or not they carry antibiotic resistance genes has become an important indicator to evaluate the safety of bacterial strains and to determine whether they can be used as a safe strain for food. In this paper, the drug resistance gene of *T. halophilic* has a high coincidence rate with the drug resistance phenotype, but it has not reached full compliance. It is necessary to continue to do more work in the detection of drug resistance genes of *T. halophilic*, such as the way in which *T. halophilic* acquire resistance, whether there is resistance gene transfer between strains, and factors affecting drug resistance gene transfer.

## 4. Materials and Methods 

### 4.1. Material

For the current study, a total of 49 *T. halophilus* strains isolated from two Xuanwei hams were used. The Xuanwei hams in this study were gathered from local markets in Kunming, Yunnan province.

The high-speed desktop centrifuge (H318K) was purchased from Hunan Kecheng equipment Co., Ltd. (Hunan, China), the Ultra-micro UV spectrophotometer (NanoDrop 2000) was purchased from Thermo Fisher Scientific (Waltham, MA, USA), and the Gel imaging system (Gel DOC XR) was purchased from Bio-Rad Laboratories (Hercules, CA, USA).

### 4.2. Xuanwei Ham Samples and Bacterial Strain Isolation

The 25 g sample was aseptically chopped and immersed in 225 mL of 0.85% sterile normal saline for 15 minutes. The same was sealed on a shaker and was shaken at 280 r/min, 4 °C for 30 min. The sample solution was diluted continuously from 10^−1^ to 10^−7^ for use.

A total of 0.1 mL of each dilution (10^−1^–10^−7^) was pour-plated with DeMan Rogosa Sharpe (MRS) medium (Oxoid, Basingstoke, UK) containing 1% (w/v) Agar, 3% (w/v) NaCl [31], and 0.2% (w/v) CaCO_3_ at 30 °C for 3–4 days under semi-anaerobic conditions. Separation and purification of bacterial colonies used the same type of agar medium incubated at 30 °C for 2 days. After purifications, bacterial isolate strains were enriched and cultured in MRS medium containing 3% (w/v) NaCl at 30 °C for 48 h. The isolated strains were preserved in a ratio of 30% glycerol to 1:1 and stored at −80 °C.

### 4.3. Identification of Isolates by groEL Gene and 16S rRNA Gene

Genomic DNA of isolates was extracted using a kit (TaKaRa MiniBEST Bacteria Genomic DNA Extraction Kit Ver.3.0). Amplification primers of the 16S rRNA gene and *groEL* gene were performed and are shown in Table 6. PCR was performed using an ABI SimpliAmp thermal cycler (Applied Biosystems) in a 25 µL reaction system containing 2.5 µL of 10 × PCR Buffer(Mg^2+^ plus), 2 µL of dNTP Mixture(2.5 mM), 1 μL of each primer (10 µM), 0.2 μL of rTaq DNA Polymerase (5 U/µL) (TaKaRa Biotechnology, Beijing, China), 2 µL of DNA template, and 16.3 µL of ultrapure water. The 16S rRNA amplification was carried out the with the following program: Initial denaturation at 94 °C for 2 min; 30 cycles of denaturation at 94 °C for 30 s, primer annealing at 54 °C for 30 s and primer extension 1 min at 72 °C; and a final extension of at 72 °C for 10 min. During groEL gene amplification, the procedures applied for 16S rRNA amplification were followed, but annealing was performed at 54 °C instead of 58 °C.

The 16S rRNA and *groEL* PCR products were further analyzed on 2% agarose gel with ethidium bromide (0.1 g/mL) and run with 1 × TAE buffer, using a 2-kb ladder Maker (TsingKe, Beijing, China) for molecular weight standards. The PCR products were purified and sequenced using a custom service provided by Sangon Co. Ltd. (Shanghai, China). The 16S rDNA sequence analysis was carried out using and Illumina Miseq Sequencing Instrument and the Miseq Reagent Kit V3. The 16S rRNA gene sequence similarities were searched and identification of these isolates was determined through a search of the GenBank DNA database using the BLAST algorithm. The phylogenetic positions of the isolates were inferred by 16SrRNA gene sequence analysis.

### 4.4. Antibiotic Sensitivity Tests of Isolates

*T. halophilus* strains were cultured twice in MRS broth containing 3% NaCl and matched to a McFarland 0.5 turbidity standard (BIO-KONT, Shenzhen, China). Antibiotic diffusion tests were determined by the Bauer−Kirby method according to the guidelines of the Clinical and Laboratory Standards Institute (CLSI). The phenotypic antimicrobial sensitivity response of each *T. halophilus* isolate was evaluated using panel of 20 antimicrobial discs. The antimicrobial agents used included 10 µg penicillin (PEN), 10 μg amoxicillin (AMO), 10 μg ampicillin (AMP), 10 μg streptomycin (STR), 10 μg gentamicin (GEN), 30 μg tetracyclines (TET), 300 μg polymyxin B (PB), 1.25 μg compound sulfamethoxazole (COM), 5 μg ciprofloxacin (CIP), 5 μg ofloxacin (OFZ), 30μg ceftazidime pentahydrate (CAZ), 30 μg ticarcillin (TIC), 30 μg chloramphenicol (CHL), 30 μg cefradine (CE), 30 μg cefotaxime sodium (CTX), 30 μg neomycin (NE), 30 μg minocycline (MH), 30 μg doxycycline (DO), and 30 μg vancomycin (VA). All the antibiotics discs were purchased from the company Oxoid (UK). Measurement of the diameter of the zone of inhibition was to the nearest millimeter. Since CLSI has not yet defined the inhibition zone diameter value of the *Tetragenococcus* genus antibiotic, according to the taxonomic position, Enterococcus is the closest to *Tetragenococcus* in phylogeny. Therefore, the *T. halophilus* isolates were classified as susceptible, intermediate, and resistant according to the definition of antimicrobial circle diameter for the genus *Enterococcus* interpretative standards of CLSI.

### 4.5. Antibiotic Resistance Gene Detection of Isolates

All primers were designed using Vector NTI Advance 10 software according the antibiotic resistance gene information provided by the Antibiotic Resistance Genes Database (ARGD) and some primers were introduced in the references and synthesized by Sangon Co. Ltd. (Shanghai, China). The detailed sequences of these primers, optimum annealing temperatures, and product size are listed in Table 7. The PCR amplification in a 25 µL reaction system contained 2.5 µL 10× Ex Taq Buffer, 2 µL dNTP Mixture (2.5 mM), 1 μL of each primer, 0.2 μL ExTaq DNA Polymerase (TaKaRa Biotechnology, Beijing, China), 2 µL DNA template, and 16.3 µL ddH_2_O.

## 5. Conclusions

We assessed the prevalence of antibiotic resistance phenotypes (K-B method) and genotypes (PCR method) of 49 *T. halophilus* strains isolated from Xuawei ham in China. The results showed that 49 isolates can be considered as completely susceptible to penicillin, ampicillin, amoxicillin, cefradine, cefotaxime, tetracyclines, minocycline, doxycycline and vancomycin, but resistant to gentamicin, streptomycin, neomycin, polymyxinB and cotrimoxazole, which were consistent with the previous reports. These resistances were sufficiently high to consider the potential for acquisition of transmissible determinants. Among the 26 antibiotic resistance genes, 5 varieties of antibiotic resistance genes, including *acrB*, *blaTEM*, *AAda1*, *SulII* and *GyrB*, were detected, and the detection rates were 89.79%, 47.7%, 16.33%, 77.55%, and 75.51%, respectively. The potential acquisition of transmissible determinants for antibiotic resistance and antibiotic resistance genes identified in this study necessitate the need for a thorough antibiotic resistance safety assessment of *T. halophilus* before it can be considered for use in food fermentation processes.

## Figures and Tables

**Table 1:** Pharmacokinetic phenotypic test results of 49 strains of *T. halophilus.*

Antibiotic	Isolates of Resistant	Isolates of Susceptible	Resistance Rate (R)		Intermediate Rate (I)		Susceptible Rate (S)	
PEN	0	49	0%	(0/49)	0%	(0/49)	100%	(49/49)
AMO	0	47	0%	(0/49)	4.08%	(2/49)	95.90%	(47/49)
AMP	0	49	0%	(0/49)	0%	(0/49)	100%	(49/49)
TIC	2	47	4.08%	(2/49)	0%	(0/49)	95.9%	(47/49)
CE	0	49	0%	(0/49)	0%	(0/49)	100%	(49/49)
CAZ	2	42	4.08%	(2/49)	10.20%	(5/49)	85.70%	(42/49)
CTX	0	49	0%	(0/49)	0%	(0/49)	100%	(49/49)
GEN	49	0	100%	(49/49)	0%	(0/49)	0%	(0/49)
STR	49	0	100%	(49/49)	0%	(0/49)	0%	(0/49)
NE	49	0	100%	(49/49)	0%	(0/49)	0%	(0/49)
TET	0	49	0%	(0/49)	0%	(0/49)	100%	(0/49)
MH	0	49	0%	(0/49)	0%	(0/49)	100%	(0/49)
DO	0	49	0%	(0/49)	0%	(0/49)	100%	(0/49)
PB	49	0	100%	(49/49)	0%	(0/49)	0%	(0/49)
VA	0	49	0%	(0/49)	0%	(0/49)	100%	(0/49)
COM	49	0	100%	(0/49)	0%	(0/49)	0%	(0/49)
CHL	4	40	8.16%	(0/49)	10.20%	(5/49)	81.63%	(40/49)
OFZ	32	2	65.31%	(32/49)	30.61%	(15/49)	4.08%	(2/49)
CIP	4	33	8.16%	(4/49)	24.49%	(12/49)	67.35%	(33/49)

**Table 2:** Drug resistance spectrum of 49 strains of *T. halophilus.*

Type of Resistance	Resistant Spectrum	Isolates
5	GEN-STR-NEO-PB-COM	49
6	GEN-STR-NEO-PB-COM-OFZ	32
7	GEN-STR-NEO-PB-COM-OFZ-CIP	4
	GEN-STR-NEO-PB-COM-OFZ-CAZ	2
	GEN-STR-NEO-PB-COM-OFZ-TLC	2
	GEN-STR-NEO-PB-COM-OFZ-CHL	2
8	GEN-STR-NEO-PB-COM-OFZ-CIP-CHL	4
	GEN-STR-NEO-PB-COM-OFZ-CIP-CAZ	1

**Table 3:** Multi-drug resistance of 49 strains of *T. halophilus.*

Type of Resistance	Number of Isolates	Proportion (%)
0	0	0
1	49	100%
2	49	100%
3	49	100%
4	49	100%
5	49	100%
6	32	65.31%
7	12	24.50%
8	5	10.20%

**Table 4:** Detection rate of 26 drug resistance genes in 49 strains of *T. halophilus.*

Antibiotic	AR Gene	Positive Isolates	Totally Isolates	Positive Rates
Tetracyclines	*tet(K)*	0	49	0%
	*tet(L)*	0	49	0%
	*tet(M)*	0	49	0%
	*tet(O)*	0	49	0%
	*tet(S)*	0	49	0%
	*tet(W)*	0	49	0%
*β*-lactams	*blaTEM*	8	49	16.33%
	*bl3-vim*	0	49	0%
	*blaOXA*	0	49	0%
	*blaSHV*	0	49	0%
Sulfonamides	*Sul1*	0	49	0%
	*Sul2*	38	49	77.55%
	*Sul3*	0	49	0%
Aminoglycosides	*aac(3′)-IIa*	0	49	0%
	*acrB*	44	49	89.79%
	*aadB*	0	49	0%
	*aadA1*	14	49	28.57%
Chloram Phenicols	*floR*	0	49	0%
	*Cat1*	0	49	0%
Quinolones	*GyrA*	0	49	0%
	*GyrB*	37	49	75.51%
	*ParC*	0	49	0%
Polypeptide Antibiotic	*VanC1*	0	49	0%
	*EmgrB*	30	49	61.22%

**Table 5:** Drug-resistant phenotype and genotype coincidence rate of *T. halophilus.*

Antibiotic	Resistant Phenotype	Resistant Gene	Compliance Rate (%)	
Tetracyclines	0	0	(0/0)	100%
*β*-lactams	5	8	(5/8)	62.5%
Sulfonamides	49	38	(38/49)	79.17%
Aminoglycosides	49	45	(45/49)	91.84%
Chloram phenicols	4	0	0	0
Quinolones	32	37	(32/37)	86.49%
Polypeptide Antibiotic	49	30	(30/49)	61.22%

**Table 6:** Primers sequences of PCR amplification.

Primers	Gene Sequences (5’–3’)	References
27-F	AGATTTGATCCTGGCTCAG	[32]
1492-R	CTACGGCTACCTTGTTACGA	
GroEL-F	CGTCGTCAATGCTYAATGG	[33]
GroEL-R	TGCTGCCAGAAGAAACTTCA	

**Table 7:** Primers sequences of PCR amplification.

Antibiotic	AR Gene	Gene Sequences (5‘–3‘)	Annealing Temp. (°C)	Product Size (bp)	Ref.
Tetracyclines	*tet(K)*	F: TTAGGTGAAGGGTTAGGTCC R: GCAAACTCATTCCAGAAGCA	59	697	[30]
	*tet(O)*	F: AACTTAGGCATTCTGGCTCAC R: TCCCACTGTTCCATATCGTCA	55	515	[30]
	*tet(S)*	F: CATTTGGTCTTATTGGATCG R: ATTACACTTCCGATTTCGG	55	456	[30]
	*tet(W)*	F: GAGAGCCTGCTATATGCCAGC R: GGGCGTATCCACAATGTTAAC	58	168	[30]
	*tet(L)*	F: TCATCATCTCCTGATTTTAC R: AGTAAAAACAAGCAGAGCAT	60	1464	This study
	*tet(M)*	F: GTTAAATAGTGTTCTTGGAG R: CTAAGATATGGCTCTAACAA	53	501	This study
*β*-lactams	*blaTEM*	F: CAGAAACGCTGGTGAAAG R: TTACCAATGGTTAATCAGTGAG	54	788	[34]
	*bl3-vimF*	F: TTGGTCTACATGACCGCGTCTGTA R: AGATCGGCATCGGCCACGTT	59	623	This study
	*blaOXA*	F: TTTTCTGTTGTTTGGGTTTC R: TTTCTTGGCTTTTATGCTTG	53	447	[35]
	*blaSHV*	F: TGTATTATCTCCCTGTTAGC R: TTAGCGTTGCCAGTGCTC	55	843	[35]
Sulfonamides	*Sul1*	F: TCGGACAGGGCGTCTAAG R: GGGTATCGGAGCGTTTGC	63	925	[35]
	*Sul2*	F: CCTGTTTCGTCCGACACAGA R: GAAGCGCAGCCGCAATTCAT	55	435	This study
	*Sul3*	F: ATGAGCAAGATTTTTGGAATCGTA R: CTAACCTAGGGCTTTGGATATTT	59	792	[36]
Aminoglycosides	*aac (3′)-IIa*	F: GGCGACTTCACCGTTTCT R: GGACCGATCACCCTACGAG	54	412	[35]
	*acrB*	F: CGTGAGCGTTGAGAAGTCCT R: GGCGTCAGTTGGTATTTGGT	58	222	[37]
	*aadB*	F: GAGGAGTTGGACTATGGATT R: CTTCATCGGCATAGTAAAA	53	208	This study
	*aadA1*	F: TTTGCTGGTTACGGTGAC R: GCTCCATTGCCCAGTCG	58	497	[36]
Chloramphenicols	*floR*	F: GAACACGACGCCCGCTAT R: TTCCGCTTGGCCTATGAG	54	601	[35]
	*Cat1*	F: AGTGGAATAACGAACGAGC R: TCAGCAAGCGATATACGCAG	57	470	This study
Quinolones	*GyrA*	F: GGTGACGTAATCGGTAAATA R: ACCATGGTGCAATGCCACCA	53	810	[35]
	*GyrB*	F: GGACAAAGAAGGCTACAGCA R: CGTCGCGTTGTACTCAGATA	53	879	[35]
	*ParC*	F: CTGGGTAAATACCATCCGCAC R: CGGTTCATCTTCATTACGAA	53	987	[35]
Polypeptides	*VanC1*	F: GGTATCAAGGAAACCTC R: CTTCCGCCATCATAGCT	55	822	[38]
	*EmgrB*	F: CCGCTGAGTAATAATCCTAT R: TACAACCAAAGACGCAAT	48	492	[39]
